# Animal Welfare and Parasite Infections in Organic and Conventional Dairy Farms: A Comparative Pilot Study in Central Italy

**DOI:** 10.3390/ani12030351

**Published:** 2022-02-01

**Authors:** Matteo Chincarini, Lydia Lanzoni, Jorgelina Di Pasquale, Simone Morelli, Giorgio Vignola, Barbara Paoletti, Angela Di Cesare

**Affiliations:** Facoltà di Medicina Veterinaria, Università degli Studi di Teramo, Loc. Piano d’Accio, 64100 Teramo, Italy; mchincarini@unite.it (M.C.); jdipasquale@unite.it (J.D.P.); smorelli@unite.it (S.M.); gvignola@unite.it (G.V.); bpaoletti@unite.it (B.P.); adicesare@unite.it (A.D.C.)

**Keywords:** sustainability, dairy cattle, organic farming, gastrointestinal nematodes

## Abstract

**Simple Summary:**

European sustainability-oriented policies aim to encourage organic (ORG) farming practices since they are considered to be more resilient than conventional (CONV) ones and to grant higher animal welfare standards. On the other hand, animals farmed organically are considered at higher risk of parasitic infections, since grazing could expose animals to higher parasite load. Considering the contrasting data present in literature, the present work aims to investigate and compare the animal welfare conditions and gastrointestinal distribution in ORG and CONV dairy farms in central Italy. Animals from ORG farms involved in this study presented significantly less skin damages in the rear legs than animals from CONV farms. No significant differences were found for any of the other welfare-related parameters and for parasite prevalence. Results highlight that ORG farming did not have a negative impact on animal welfare and that pasture access, provided in ORG farms, did not negatively impact parasite prevalence.

**Abstract:**

The study investigated and compared welfare conditions and gastrointestinal (GI) parasites distribution among organic (ORG) and conventional (CONV) farms in central Italy. Five ORG and five CONV farms were assessed for animal welfare with an adapted version of the AssureWel protocol. Faecal samples collected from the rectum of the animals both in ORG (*n* = 150) and CONV (*n* = 150) were analysed using conventional copromiscroscopy. The presence of skin damages in the rear legs was significantly predominant (*p* < 0.001) in CONV (26.7%) compared with ORG farms (10.0%). No differences were found for lameness, cleanliness, Body Condition Score, hair loss, body lesions and swelling prevalence. Data concerning the productive performances, e.g., total milk, fat and protein yields standardised in mature equivalent (ME) were collected. ME milk yield (ORG: 9656.9 ± 1620.7 kg; CONV: 12,047.2 ± 2635.3) and ME fat yield (ORG: 396.6 ± 66.8; CONV: 450.3 ± 102.8) were significantly lower in ORG farms (*p* < 0.001). Anthelmintics were used regularly in 4/5 CONV and 0/5 ORG farms. In 2 CONV farms (40%) and 4 ORGs (80%) at least one animal tested positive for GI parasites. No significant differences in parasites prevalence emerged (ORG = 10.7%; CONV = 8%). These data indicate that ORG farming does not influence parasite prevalence and animal welfare status.

## 1. Introduction

Some of the actions promoted by the European Green Deal [1], and in particular by the “Farm to Fork” strategy [2], aim to reduce inputs in primary production. Reduction of chemical pesticides by 50%, reduction of fertilizer by 20% and reduction of sales of antimicrobial molecules and their use in animal husbandry, including aquaculture, by 50% within 2030 are encouraged. An increase in the European Utilised Agricultural Area dedicated to Organic will also be encouraged, with the aim of increasing it by 25% within 2030. Particularly, the Italian organic market is a growing sector as shown by the national registers where it has been reported that the consumption of organic products has tripled over the last 10 years. Particularly, an increase of 2.3% in the selling of dairy products was observed between 2019 and 2020 [3].

As for all European Countries, the Italian, organic production is regulated by the European Council Regulation No. 2018/848 [4], that defines several differences between organic (ORG) and conventional (CONV) farm systems [5]. ORG dairy cattle farming is characterised by high-roughage diets based on home-grown feedstuffs, outdoor access on pasture and restrictive use of antibiotics. Furthermore, ORG farms are considered to improve animal welfare compared with CONV farms and to be more respectful of the environment [6,7], even if contrasting data are available in literature. For instance, ORG farming, which has to guarantee by law the access of animals to pasture, is often perceived as more animal friendly than CONV farming [8]; also, this farming system favors aspects related to the concept of naturalness and life quality of the animals [9]. Nevertheless, little evidence is available on a higher standard of animal welfare in ORG vs. CONV farms to date [10,11].

Parasitic infections are a critical health-related issue of ORG farming, since grazing may expose animals to high parasite load [11]. The annual cost ascribed to helminth infections in dairy cattle and livestock industries in Europe is estimated to be €941 million and €1.8 billion, respectively [12]. Helminthoses have a negative impact on feed intake, growth, productivity, reproductive standards, animal health and welfare, and may increase greenhouse gas emission associated with ruminant productions [13]. Gastrointestinal nematodes (GIN) and liver flukes are considered to be a major constraint among helminths affecting cows. The control of GIN in conventional (CONV) dairy farms relies primarily on the use of anthelmintic products. This approach is increasingly unsustainable as anthelmintic resistance (AR) is spreading and it is predicted to grow in the next future [13,14]. On the other hand, the control of GIN is challenging in ORG farms where cattle are grazed and the use of anthelmintic is limited. The correct and sustainable management of GIN is therefore pivotal to increase the profitability of both ORG and CONV cattle industries.

Responding to the European Green Deal indications, increasing organic farming compared to the conventional one, could represent a problem for animal health and in particular for the management of parasitosis. Under this perspective, this case study was carried out to better understand and compare the animal welfare conditions and GIN distribution in ORG and CONV farms.

## 2. Materials and Methods

### 2.1. Farm Selection

Ten dairy farms located in central Italy were included in the study during February–March 2020. Five of the farms were managed conventionally (CONV) while the other five followed the rules for organic (ORG) production [Reg. (UE) 2018/848] [4] and were required to be licensed by an organic certifying agency. Conventional herds were identified by obtaining a list of farms from the Italian Breeders Association (AIA) and organic farms from a central database. The herd size of the farms included in the study was representative of the Italian average number of lactating cows (Italian Friesian Holstein) both for conventional (110 head/farm) [15] and organic farms (48 head/farm) [16]. A sample of 30 lactating cows was randomly selected from each farm (i.e., 150 from 5 ORG and 150 from 5 CONV farms). The average parity order of the selected animals was 2.2 ± 1.4 (mean ± standard deviation). ORG farms included in the study were provided with native grassland and surface cultivated land grazing areas nearby the stables. Animals were free to access pasture whenever weather conditions allowed and housed in the stall during the night. ORG farms were located in mid-hill areas of the central Apennines, characterised by wet pastures during the winter season. CONV farms didn’t provide pasture access to animals. All the cows both in CONV and ORG farms were housed in free range stalls and had access to a resting area provided with straw-bedded cubicles. Free stall alleys were cleaned once a day using scrapers while the straw bedding was replaced whenever needed. Animals in CONV farms were fed with Total Mixed Rations (TMRs) without limitation in the forage-concentrate ratio. On the contrary, cows in ORG farms were provided with TMR composed by at least the 60% (dry matter) of roughage, beside grazing.

At least one anthelmintic treatment per year was performed in CONV farms selected for the present study while cows in ORG farms were not treated with anthelmintic compounds. None of the farmers performed regular monitoring of endoparasites.

### 2.2. Welfare Assessment

Welfare evaluation was performed by a veterinarian experienced with farm animals, using a modified version of the AssureWel protocol [17]. The following measures were assessed individually and scored as reported in Table 1 (please see the guidelines at: http://www.assurewel.org/dairycows.html; accessed 10 December 2020). Animals were kept at the self-closing feeding rack during the evaluation period. The body condition score (BCS) was evaluated observing the animal from behind and from the side. At the same time, recording of cleanliness of hind quarters and udders was performed. Finally, the type and the region of any skin damage was recorded. Afterwards, cows were individually released for the mobility assessment. For this last evaluation, each cow was observed from the side and the rear, while making between 6–10 uninterrupted strides. Furthermore, the number of broken tails were recorded. After the assessment of the last animal, the farmer was asked to walk for five minutes in the barn. During this period, the response of the cattle to the stockperson (possible options were: sociable, relaxed or nervous) was evaluated. Finally, from the farm registers the number of mastitis per 100 cows in the last 12 months were recorded.

The assessment started in the morning, two hours after milking and took no more than 45 min for each farm. Since housing conditions can influence legs and locomotion problems [18,19] only farms with cubicle housing were selected.

### 2.3. Milk Yield and Quality

Data on the productive performances were obtained from the regional breeders association (AIA) recording, choosing the official monitoring closer to the farm visit. Specifically, the following characteristics of milk variables standardised by AIA in mature equivalents have been evaluated per cow: (i) total milk yield in kg (ME milk), (ii) total fat yield in kg (ME fat), (iii) total protein yield in kg (ME protein). This standardisation represents the individual cumulative 305-day production adjusted for age and month of calving. Moreover, fat and protein percentage were calculated from the ME data.

### 2.4. Parasitological Sampling and Analysis

A fecal sample was collected from the rectum of each cow. All samples were placed in individual bags, identified, stored at refrigeration temperatures (4 °C) and transported to the Laboratory of Parasitology and Parasitic Diseases of the University of Veterinary Medicine of Teramo for examination.

All the samples were analyzed within 24–48 h using conventional copromicroscopic techniques. Specifically, all the faecal samples were examined by qualitative flotation with zinc sulfate (p.s. 1350) solution and by sedimentation method [20,21]. Stool samples were also tested using a modified McMaster technique, with a sensitivity of 50 oocysts per gram of faeces (OPG)/eggs per gram of faeces (EPG) [20].

Parasitic elements were identified based on morphological and morphometrical characteristics by light microscopy, under 100× or 400× magnification [22,23].

### 2.5. Statistical Analysis

Data collected for the welfare assessment were grouped for the statistical analysis.

The mobility score variable was grouped in two levels (score 1, 2 and 3 grouped as lame; score 0 considered as not lame) as well as the cleanliness score variable (clean: score 0; unclean: score ≥ 1). The same criteria were used to pool the presence of swellings, lesions, or hair loss in the different areas of the body (presence or absence of the alteration) and considered as “skin damage”. After a preliminary exploration of the data, the lesions affecting the head, the neck, the body, and the front legs were combined in a single variable while the region of the rear legs was kept as a separate variable.

The variables referred to parasitic infections were grouped into presence/absence of endoparasites (i.e., positive/negative to at least one endoparasite) and into presence/absence of Strongilidae, Coccidia and *Trichuris* spp. (i.e., positive/negative to at least of them).

The effect of the farming system (ORG and CONV) on animal welfare and parasite infection was analysed with Pearson’s Chi-squared test (when *p* < 0.05 Cramer’s V effect size has been reported). Moreover, the same variables were analysed with Pearson’s Chi-squared test to evaluate the effect of parity order. All analyses have been performed with R [24].

A logistic model (estimated using ML) was fitted to predict mobility score with farm type, cleanliness, head, and body skin damage (indicated as BodySkinDamage), Rear Leg Skin damage and a new BCS score (considering BCS < 2 as “thin body condition” and values 2:3 as “normal BCS”). Model fit was then evaluated visually through binned residual plot [25].

Differences in milk production (ME milk yield in kg) and quality (ME milk fat and protein in kg) were evaluated with multivariate analysis of variance after checking for: normality, multicollinearity, and homogeneity of covariances. Follow-up Welch *t*-test was performed on the dependent variable. Fat and protein percentages, calculated from the ME data, were analysed using Welch *t*-test.

## 3. Results

### 3.1. Welfare Assessment

No broken tails were registered and the response of cattle to stockperson was always recorded as either “sociable” or “relaxed”.

The differences in the variables related to animal welfare between the two farming systems (ORG vs. CONV) are reported in Table 2.

No significant differences were identified between the ORG and CONV farms for lameness, cleanliness, Body Condition Score (BCS) and head and body damage prevalence.

The presence of skin damages in the rear legs differed by farm type since 26.7% of the animals in the CONV farm presented at least one lesion in the rear legs, against the 10.0% of the ORG farms (X^2^(1) = 12.9; *p* < 0.001; Cramer’s V = 0.21).

**Figure 1:**
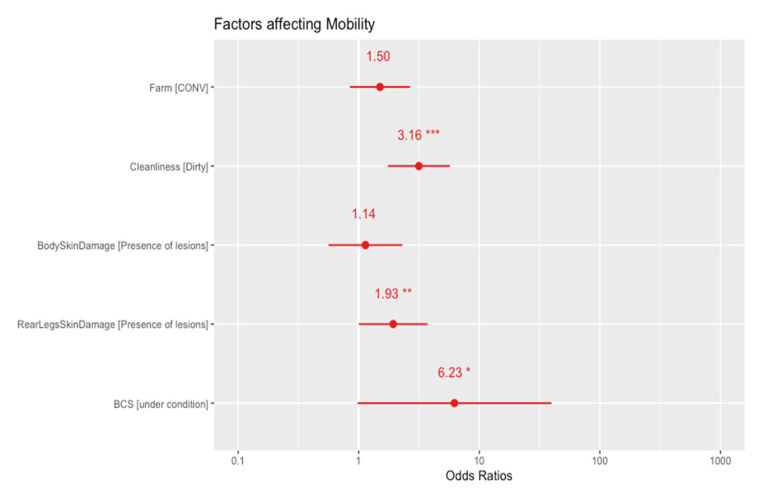
Odds-ratios from logistic regression of factors influencing the mobility scores, 95% C.I (* *p* = 0.053, ** *p* < 0.05, *** *p* < 0.01).

Considering the results from logistic regression (Figure 1) conditions that were found positively associated with impaired mobility were uncleanliness (log-odds = 1.15, 95% CI [0.56, 1.74], *p* < 0.001), presence of lesions in the rear legs (log-odds = 0.66, 95% CI [−9.17 × 10³, 1.31], *p* = 0.049) and a BCS with under condition score (log-odds = 1.83, 95% CI [−0.03, 3.90], *p* = 0.053).

The number of recorded cases of mastitis per 100 cows for the previous 12 months for ORG and COV farms were 3.6 ± 2.9 and 10.8 ± 4.1, respectively. These differences were not found to be statistically significant at the Pearson’s Chi-squared test.

### 3.2. Milk Yield and Quality

ME protein Kg (CONV = 378.7 ± 80.7; ORG = 299.3 ± 49.9), was removed from the analysis because of the high correlation with ME milk Kg (r^2^ > 0.8) and thus due to possible problems with multicollinearity in the model. A significant difference was found between the ORG and CONV farms for ME milk (kg) and ME fat (kg) (*p* < 0.001).

No statistically significant differences were found at the Welch-t test for protein percentage by farm type whereas fat percentage was higher in ORGs than in CONV farms (*p* < 0.001).

**Figure 2:**
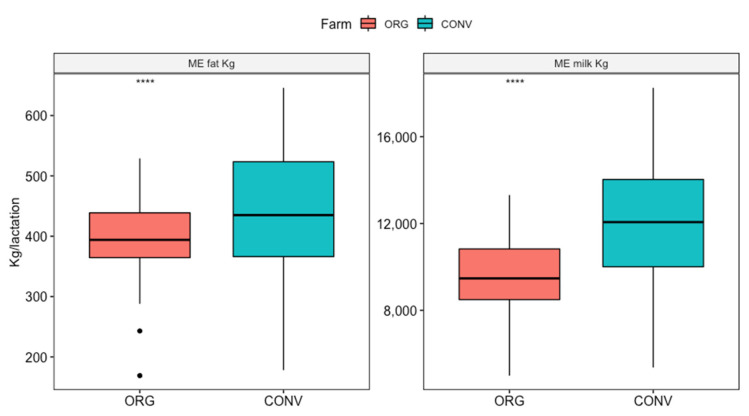
Comparison of milk production (fat and yield in kg) in the different farms (organic and conventional), ME = mature equivalent. ****: *p* ≤ 0.0001, *t*-test as Welch *t*-test; Cohen’s d effect size: (0.62 for ME fat Kg, 1.01 for ME milk Kg).

Differences in milk production and quality between the two farming systems have been reported in Table 3 and in Figure 2.

### 3.3. Parasitological Assessment

At least one animal tested positive for endoparasites in 4/5 ORG (80%) and 2/5 CONV (40%) farms, respectively. Overall, at least one parasite was found in 10.7% and 8% cows from ORG and CONV farms, respectively (Table 4).

Strongylidae eggs (ORG = 4%; CONV = 5.3%) were found in animals from both ORG and CONV farms with no significant statistical differences. *Trichuris* spp eggs were detected in 1 (0.6%) cow from a CONV farm. *Eimeria* spp. oocysts were also found in 6.7% and 5.3% cows from ORG and CONV farms, respectively. Eggs of rumen and liver flukes were not found at the sedimentation test. No significant differences in OPG/EPG values were identified between ORG and CONV farms.

## 4. Discussion

### 4.1. Welfare Assessment

Different welfare indicators have been evaluated, and among these, some differences between the two farming systems were found.

The prevalence of lameness herein obtained is similar to one of the other studies conducted in ORG farms [26] and in CONV farms using cubicle housing [27]. However, no statistically significant differences between farm type were identified in the present study in accordance with the results of Bergman et al. [28]. Other scientific papers have shown that organic herd management system is associated with a reduction of the incidence of lameness [19,29,30,31]. This is likely due to the access to pasture, which in ORG farms must be granted by law [4], that can prevent lameness in animals [32,33,34]. To support this, Silva et al. [35] reported a low prevalence of lameness in cows that had access to pasture on both CONV and ORG farms.

In the present study, animals with BCS < 2 were associated with an increased risk of mild or severe lameness (Figure 1) [36,37,38]. Accordingly, a low BCS could be considered a cause of lameness, as it is related to the thickness of the digital cushion [39,40]. On the other hand, lame animals could be less able to compete with sound herd mates for food intake, and this, when prolonged, can determine a low body condition in affected animals [41]. Therefore, it is still not clear whether a low BCS may be the cause of higher lameness prevalence or vice versa. The link between the presence of rear legs lesions and lameness has been previously associated with the floor type (if the concrete is either damaged or eroded), the automatic scraper and the presence of pushing or sharp turning near the parlor exit or entrance [29]. In this study, unclean animals were more prone to lameness. This is in line with the results of previous studies demonstrating that infectious lameness could be attributed to an unhygienic environment [42,43].

No statistically significant differences were identified between ORG (with pasture access) and CONV (without pasture access) farms in terms of animal cleanliness. Body dirtiness increases the risk of developing health-related issues such as mastitis [44,45] and foot problems [46], with a negative impact on productivity. Animal cleanliness can be influenced by several factors such as stall design [27], lying surface properties, use of bedding [47], herd size [48] and pasture access [49,50]. However, restraining animals from grazing should not be considered the only solution to avoid uncleanliness [51]. Indeed, the season and environmental and weather conditions are the main factors influencing animal cleanness [51]. For this study, the data collection was performed in February–March, i.e., during a season generally associated with high precipitation and wet pastures in central Italy. Thus, the absence of statistically significant differences between ORG and CONV farms herein obtained confirms that animal dirtiness cannot be prevented by only restraining animals from grazing.

The data reported in this study suggest that ORG farming has a beneficial impact on rear leg conditions, as lesions in organically managed animals were significantly less frequent than in CONV farms, in accordance with the results of other studies [27,28,52,53,54]. An explanation for this association could be due to the pasture access guaranteed to ORG farmed animals, as the latter has been reported to be correlated with lower hock and knee lesions [32,55,56]. Although bedding conditions were similar among farms included in the present study (all farms provided straw-bedded cubicles), it should be taken into account that the presence of lesions in these regions, especially on the hock, is strongly indicative of uncomfortable resting areas (i.e., inappropriate cubicle dimensions, the abrasiveness or hardness of the lying surfaces and improper bedding quality) [57]. It is worthy to underline that skin damages or alterations reflect an impairment of cow’s welfare since they may be painful for the animals themselves and could be the result of repeated conflicts between cows (hierarchy) and their environment [27].

BCS is an assessment of the proportion of body fat, and it is included within welfare indicators for dairy cows because it is known to be associated with animal health, reproduction, productivity and historical levels of feed intake, as reviewed by Roche et al. [58]. The present results show that the BCS of animals was not affected by farm type (i.e., CONV versus ORG), in accordance with previous reports [5,59,60,61]. BCS is associated with the correct balance of the feeding requirements [62]. A major concern in ORG farms regards the risk of severe negative energy due to the limited proportion of concentrate feed related to the legislation [Reg. (UE) 2018/848] [4,5,58,59,60]. Furthermore, nutritional deficiencies may also occur in certain areas where the pasture is poor [10]. 

The results of the present study showed no differences between the farming systems in terms of mastitis. While the sample size was limited, the present findings are similar to other studies [63,64]. Although the literature shows controversial results, some evidence supports that cow managed in ORG systems are less affected by mastitis [35,51]. One of the major limits is explained by differences in the study design where mastitis are considered either only as clinical or as sub-clinical too. This latter implying the presence of bacteria and the increase of somatic cell count (please, for a detailed review see also Åkerfeldt et al. [10]).

### 4.2. Milk Yield and Quality

As expected, the present data show that milk production from ORG reared cows is affected by a reduction in terms of milk yields [65,66,67]. In an exhaustive review by Van Wagenberg et al. [68], the authors found that, despite the lower milk yield production by ORG farms, these latter had farm-gate price premiums up to 84% above the conventional prices. Still, variable costs and total costs per cow were lower, ending in higher farming income per animal. However, economical comparisons between ORG and CONV in dairy cattle are still controversial and need to be further addressed [68].

Contrasting results can be found in the literature for fat and protein content [68,69]. Particularly, Adler et al. and Rodríguez–Bermúdez et al. [70,71] found lower milk production in ORG farms but no differences in protein and fat percentage. Mullen et al. [63] found no differences in any of the parameters, while in contrast, Vicini et al. [72] found a higher protein content. No significant differences were found in terms of protein percentage, in agreement with Butler et al. [73]. On the other hand, a higher percentage of fat was found in milk from ORG farms in accordance with Butler et al. and Kouřimská et al. [73,74]. This difference could be attributable to several factors. For instance, feeding strategies, with access to grazing areas and at least 60% (dry matter) of roughage in daily ration, as stated by the EU legislation for ORG farms [4], could represent the main effective factor [64,75].

### 4.3. Parasitological Assessment

Organic dairy farming systems have been traditionally associated with an increased risk of exposure to a range of pasture born parasites, such as GIN [76,77]. Moreover, cattle grazing and fed with fresh grass are more likely to be infected with rumen (e.g., *Calicophoron daubneyi*) and liver (e.g., *Fasciola hepatica*) flukes [78]. Data herein generated demonstrated that there are no significant differences in GI helminthes infections between the two farming systems. These findings are in line with those of other studies showing that in the same geographical areas the prevalence of GIN infections in ORG farms may be similar, or lower, than that reported in CONV ones [79,80]. Tolerance to GIN may be indeed higher in livestock reared on ORG farms due to the continuous exposure to parasite infections that may lead to improved resilience [79]. The life cycle of rumen and liver flukes requires freshwater gastropods (e.g., different species of Lymnaeidae) which ensures the development of the parasites. Accordingly, their geographic distribution is not homogenous and it is strictly influenced by environmental conditions. On the other hand, the absence of Trematoda in the animal population examined in the present study should be evaluated with caution, considering that the diagnostic sensitivity of copromicroscopy after sedimentation technique is below 100% [81].

Despite the small sample size, helminthes and coccidian infections were present in cattle from both ORG and CONV farms, although with an apparent low infection rate. These infections are a potential threat for animal welfare and may cause relevant economic losses. Therefore, further studies are warranted to investigate the distribution of these parasites in a higher number of farms and animals, including both adults and replacement stocks. The actual impact of parasitism on the animal health, welfare and production should also be considered. Specific and sustainable control programs, planned according to different species circulating at the farm level, should be evaluated.

## 5. Conclusions

In line with the study objective, the similarities and differences between CONV and ORG farming systems in central Italy have been described. In this case study a limited number of farms were assessed for animal welfare, productivity and parasite prevalence. On the whole, allowing animals to pasture, as is requested for ORG farming, did not have a negative impact on animal welfare in the study population, confirming the value on animal welfare desired by the legislation. This was also highlighted by the little differences in parasite prevalence that should orient future research on integrated control measures and targeted selective treatments, also to improve organic production and better respond to the European policy of the European Green Deal [1] and the “Farm to Fork” strategy [2]. Further studies which include a larger number of farms could investigate those topics in more detail.

## Figures and Tables

**Table 1:** Measures collected for animal welfare assessment (adapted from AssureWel [17]).

Measures	Description	Score
Mobility	Good/Imperfect	0/1
	Impaired	2
	Severely Impaired	3
Body Condition Score	Thin	1
	Moderate	2–3
	Fat	4–5
Cleanliness	No dirt/only minor splashing	0
	Very dirty: plaques > forearm length	2
Hair Loss, Lesions and Swellings *	No/slight skin damage/swelling	0
	Hairless patch (1 or more ≥ 2 cm)	H
	Lesion (1 or more ≥ 2 cm)	L
	Mild swelling	1S
	Substantial swelling (≥5 cm in diameter)	2S
Response of cattle to stockperson	Sociable	0
	Relaxed	1
	Nervous	2
Broken tails	Number of broken tails observed	
Mastitis	Number of recorded cases of mastitis per 100 cows for the previous 12 months.	

* Evaluated on head and neck, body, front legs and rear legs.

**Table 2:** Results of welfare assessment on animals housed in organic (ORG) and conventional (CONV) farms. *n*/tot = number of records on the total of assessed animals; % = percentage; mean ± sd is referred to single farms within farm type.

	ORG		CONV	
Welfare Measures	*n*/tot (%)	Mean ± s.d./Farm	*n*/tot (%)	mean ± s.d./Farm
**Lame animals**	29/150 (19.3)	5.8 ± 2.6	41/150 (27.3)	9 ± 2.4
**Unclean animals**	44/150 (29.3)	8.8 ± 2.2	29/150 (19.3)	5.8 ± 1.1
**Animals with rear leg skin damage**	15/150 (10.0) ^B^	3 ± 0.7	40/150 (26.7) ^A^	8 ± 2.0
**Animals with head and body damage**				
Total	27/150 (18.0)	5.4 ± 1.1	31/150 (20.7)	6.2 ± 1.9
Animals with head and neck skin damage	5/150 (3.3)	1 ± 0.7	5/150 (3.3)	1.2 ± 1.1
Animals with body skin damage	7/150 (4.7)	1.4 ± 1.1	11/150 (7.3)	2 ± 1.2
Animals with front legs skin damage	15/150 (10.0)	3 ± 0.7	14/150 (9.3)	3 ± 0.7
**BCS**				
1	1/150 (0.7)	0.2 ± 0.4	4/150 (2.7)	0.8 ± 0.4
2	34/150 (22.7)	6.8 ± 1.9	35/150 (23.3)	7 ± 1.6
3	85/150 (56.7)	17 ± 3.2	86/150 (57.3)	17.2 ± 4.1
4	30/150 (20.0)	6 ± 1.6	25/150 (16.7)	6 ± 2.3

Means with different superscript (^A,B^) letters were found statistically different with *p* < 0.001.

**Table 3:** Milk quantity and quality by farm type. Means with different superscript (^A,B^) letters were found statistically different with *p* < 0.001 at Welch *t*-test. Results are reported as mean ± standard deviation.

Milk Yield and Quality	CONV	ORG
ME milk (kg)	12047.2 ± 2635.3 ^A^	9656.9 ± 1620.7 ^B^
ME fat (kg)	450.3 ± 102.8 ^A^	396.6 ± 66.8 ^B^
Fat (%) *	3.7 ± 0.6 ^B^	4.1 ± 0.6 ^A^
Protein (%) *	3.1 ± 0.4	3.1 ± 0.2

* Fat and protein percentage were calculated from the Mature Equivalent data.

**Table 4:** Results of the copromicroscopic examinations. Percentage (%) of positive cows and minimum and maximum OPG (oocysts per gram of faeces)/EPG (eggs per gram of faeces). All samples tested negative at the sedimentation technique.

	ORG		CONV		TOTAL
	Flotation	McMaster	Flotation	McMaster	Flotation
	*n*/tot (%)	OPG/EPG	*n*/tot (%)	OPG/EPG	*n*/tot (%)
**Coccidia**	10/150 (6.7)	<50–150	8/150 (5.3)	<50–150	18/300 (6)
**Strongilidae**	6/150 (4)	<50–50	8/150 (5.3)	<50	14/300 (4.7)
***Trichuris* spp.**	0	-	1/150 (0.7)	<50	1/300 (0.3)
Positive * cows	16/150 (10.7)	-	12/150 (8)	-	28/300 (9.3)
**Negative cows**	134/150 (89.3)	-	138 (150/192)	-	272/300 (90.7)

* Animals infected by one or more parasites.

## Data Availability

The data presented in this study are available on request from the corresponding author.
